# The Provision of Mental Health Care for Service Users With Complex Needs Who Are at Risk of Suicide

**DOI:** 10.1192/bjo.2022.241

**Published:** 2022-06-20

**Authors:** Pooja Saini, Anna Balmer, Laura Sambrook, Hana Roks, Jason McIntyre, Antony Martin, Jackie Tait, Peter Ashley-Mudie, Amrith Shetty, Rajan Nathan

**Affiliations:** 1 Liverpool John Moores University, Liverpool, United Kingdom; 2 QC Medica LLP, York, United Kingdom; 3 Cheshire and Wirral Partnership NHS Foundation Trust, Chester, United Kingdom

## Abstract

**Aims:**

Individuals presenting with complex behavioural and mental health needs may not receive the provision of care needed. Those presenting with a more complex clinical presentation may have a history of self-harm and suicide attempts. A common risk factor for preceding suicide is previous self-harm, suicide attempts or discharge from inpatient units. Understanding the descriptive symptom domains for inpatients and those treated in the community and the relationship between them could inform suicide prevention. The aim of this study was to explore the extent of self-harm and suicidal behaviours in individuals with complex mental health needs across inpatient and community settings.

**Methods:**

A cohort study design of in-depth written medical notes (n = 80) for people who were known to have complex mental health needs across inpatient and community settings. Data were extracted from medical records onto a coproduced questionnaire. As well as demographic data, information was collated about previous self-harm, suicide planning, suicide attempts, and support seeking regarding suicidal thoughts. The study will include a quantitative in-depth description and inferential analysis of the demographic clinical characteristics of the patient group.

**Results:**

Medical case notes were reviewed for 80 service users with complex mental health needs. Across both groups, approximately three-quarters of participants had previously self-harmed (76%), or planned suicide (n = 73%), and/or attempted suicide (63%). Self-harm (83% vs. 70%) and suicide attempts (72% vs. 65%) were more prevalent in the inpatient group compared to the community group. Social support was received more by community patients than inpatients (70% vs. 50%), even though inpatients were more likely to sough help when experiencing suicidal thoughts compared to people cared for in the community (38% vs. 30%). In both groups, there were often multiple suicide plans and attempts made over their timeline of contact with services.

**Conclusion:**

Self-harm, suicide planning, and suicide attempts were prevalent for people treated across both inpatient and community settings. Self-harm and suicide planning was indicative of a later suicide attempt within both settings. In those experiencing suicidal thoughts, few had sought help, suggesting the importance in staff training to enable then to recognise and identify patterns of self-harming and suicidal behaviours in individuals with complex mental health needs. Social support needs for inpatients should be increased, particularly when they sought help for suicidal thoughts. This may help to reduce length of stays in hospital or future readmissions to hospital; thus, reducing the cost implications for the NHS mental health services.

